# Altered gp130 signalling ameliorates experimental colitis via myeloid cell-specific STAT3 activation and myeloid-derived suppressor cells

**DOI:** 10.1038/srep20584

**Published:** 2016-02-05

**Authors:** Jan Däbritz, Louise M. Judd, Heather V. Chalinor, Trevelyan R. Menheniott, Andrew S. Giraud

**Affiliations:** 1 Gastrointestinal Research in Inflammation and Pathology, Infection and Immunity Research Theme, Murdoch Children’s Research Institute, The Royal Children’s Hospital Melbourne, Parkville, Victoria, Australia; 2 Department of Paediatrics, University of Melbourne, Melbourne Medical School, Parkville, Victoria, Australia

## Abstract

STAT3 regulates the expansion of myeloid-derived suppressor cells (MDSCs) during inflammation, infection and cancer. Hyperactivation of STAT3 in gp130^757F/F^ mice is associated with protection from experimental colitis. This study determined mechanisms for this protection and compared this to mice with myeloid-specific STAT3-deficiency (LysMcre/STAT3^flox^; gp130^757F/F^ LysMcre/STAT3^flox^). Acute and chronic colitis was induced and colons were removed for histological, mRNA and protein analysis. Cell populations from spleen, mesenteric lymph node and colon were analyzed for different myeloid cell populations using flow cytometry. Functions of MDSCs and LPS-stimulated peritoneal macrophages were further characterized by *in vitro* and *in vivo* assays. Here we show that the resistance to experimental colitis in gp130^757F/F^ mice is via myeloid-cell specific STAT3 activation, MDSC expansion and increased production of suppressive and protective cytokines.

Myeloid-derived suppressor cells (MDSCs) are a heterogeneous population of cells of myeloid origin that comprises myeloid progenitor cells and immature myeloid cells^1^. They expand during inflammation, infection and cancer and are potent suppressors of various T-cell functions^2^,^3^. In addition, MDSCs regulate innate immune responses and non-immunological processes^4^,^5^. In mice, MDSCs consist of two main subsets: monocytic (M-)MDSCs, which have a CD11b^+^Ly6G^–^Ly6C^high^CD49^+^ phenotype, and granulocytic (G-)MDSCs, which have a CD11b^+^Ly6G^+^Ly6C^low^CD49^–^ phenotype^6^,^7^. Most of the factors that induce MDSC expansion and activation trigger signalling pathways in MDSCs that converge on Janus kinase (JAK) protein family members, signal transducer and activator of transcription (STAT), extracellular signal regulated kinase (ERK) and nuclear factor-κb (NFκB), which are signalling molecules that are involved in cell survival, proliferation and differentiation^8^,^9^. The M-MDSC subset shows upregulated expression of STAT1, whereas G-MDSCs are characterized by increased activity of STAT3. Abnormal and persistent activation of STAT3 in myeloid progenitor cells prevents their differentiation into mature myeloid cells and thereby promotes MDSC expansion. Activation of both MDSC subsets in pathological conditions results in increased levels of arginase 1, which is an important immune suppressive factor^4^. In addition, MDSCs induce a T helper cell type 2 (Th2) phenotype by producing the Th2 cell cytokine interleukin (IL)-10^10^.

Emerging evidence suggests that MDSCs play a regulatory role in inflammatory bowel diseases (IBD), in which an abnormal immune response against the microorganisms of the intestinal flora and a breakdown in diverse regulatory pathways is responsible for the chronic inflammatory pathology in genetically susceptible individuals^11^,^12^. The adoptive transfer of MDSCs in different animal models of IBD ameliorates colitis, and suggest that MDSCs may be used as the basis for a novel adoptive cell therapy in IBD^13^,^14^,^15^,^16^,^17^. However, the mechanisms underlying the influence of MDSCs on inflammatory responses in the pathogenesis of IBD remain elusive^5^,^18^.

The signalling of immune system mediators plays a key role in diseases of the gut. This includes the glycoprotein (gp)130 receptor, which binds the diverse, 10 member IL-6 family of cytokines and signals through STAT1/3 and RAS/ERK^19^,^20^. Mice with a myeloid-specific defect in STAT3 (LysMcre/STAT3^flox^) develop chronic colitis secondary to the inability of myeloid cells to respond to IL-10, and is dependent on the interaction with lymphocytes^21^,^22^. Mice with mutations that abrogate gp130-induced STAT1/3 signalling have an increased susceptibility to experimentally-induced colitis. In contrast, gp130 ‘knock-in’ mutant mice (gp130^757F/F^) which are incapable of activating the RAS/ERK pathway and have hyperactivation of STAT3 in response to gp130 engagement, are protected from chemically induced colitis using dextran sulphate sodium (DSS)^23^,^24^,^25^.

We hypothesized that the protective role of gp130-dependent STAT3 activation in experimental IBD involves the expansion and activation of MDSCs, in addition to the previously reported proliferative, regenerative and survival effects on intestinal epithelial cells^24^,^25^,^26^. In the present study we therefore analyzed the immunoregulatory role of the transcription factor STAT3 in experimental mouse models of IBD using gp130^757F/F^ mice with systemic hyperactivation of STAT3; mice with a myeloid-cell specific deficiency of STAT3 (LysMcre/STAT3^flox^); and mice with systemic hyperactivation of STAT3 with a myeloid-cell specific deficiency of STAT3 (gp130^757F/F^ LysMcre/STAT3^flox^). Our results show that the resistance to acute DSS-induced colitis in gp130^757F/F^ mice occurs via myeloid-cell specific STAT3 activation, expansion of granulocytic MDSCs in the colon and increased production of suppressive and protective mediators. Mice with myeloid-specific STAT3-deficiency were not protected from DSS-induced colitis, which was associated with impaired expansion of mucosal MDSCs and reduced production of anti-inflammatory cytokines. Thus, our study identifies new immunoregulatory mechanisms of STAT3 during intestinal inflammation.

## Results

### gp130^757F/F^ mice are resistant to acute but not chronic DSS-induced colitis

**Figure 1:**
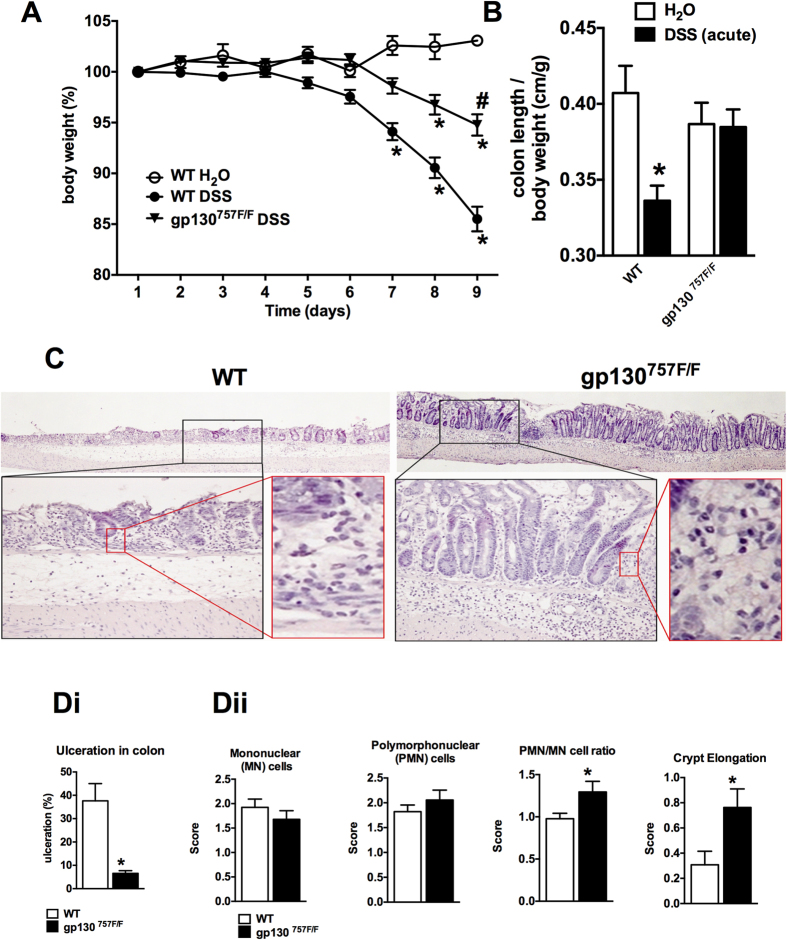
Acute colitis susceptibility in gp130^757F/F^ mice. **(A)** Acute colitis was induced in wild type (WT) mice (n = 20) and gp130^757F/F^ mice (n = 20) with 3% DSS in drinking water and compared with untreated WT mice (H_2_O, n = 20). The daily changes in body weight were calculated over the course of at least three independent experiments. *indicates *P* < 0.05 compared to mice receiving only water; ^#^indicates *P* < 0.05 in mice of different genotype both receiving DSS. **(B)** On day 9 colons were removed and the colon length per body weight (±SEM) of each experimental group is shown. **(C)** Microscopic analysis of H&E-stained colon sections was performed and representative images demonstrating ulceration, inflammation and crypt elongation are shown. **(Di)** The proportion of colon ulcerated relative to the whole colonic mucosa was calculated. **(Dii)** Semi-quantitative histological comparative analysis of WT and gp130^757F/F^ mice was performed. For B & D bars refer to mean (±SEM). *indicates *P* < 0.05 compared to control.

**Figure 2:**
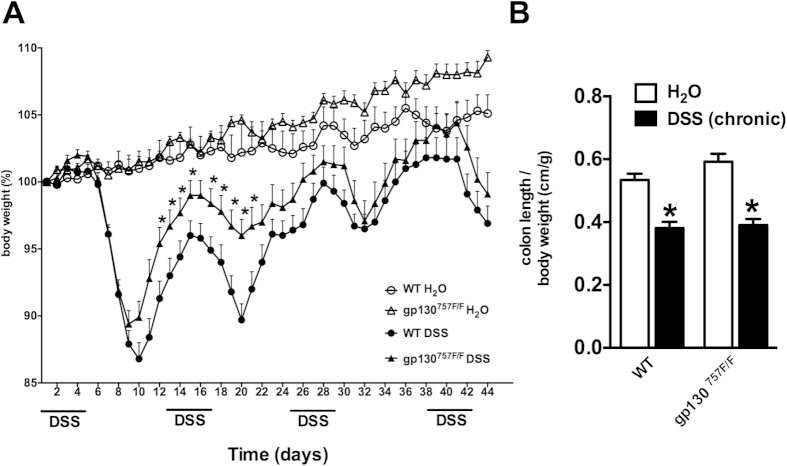
Chronic colitis susceptibility in gp130^757F/F^ mice. **(A)** Chronic colitis was induced in wild type (WT) mice (n = 20) and gp130^757F/F^ mice (n = 20) with 3% DSS in drinking water on the days indicated and compared with untreated mice (H_2_O, n = 20 in each group). The daily changes in body weight were calculated over the course of at least three independent experiments. *indicates *P* < 0.05 in mice of different genotype both receiving DSS. **(B)** The graph shows the mean colon length on day 44 (chronic DSS-induced colitis) per body weight (±SEM) of each experimental group. *indicates *P* < 0.05 compared to wild type.

gp130^757F/F^ mice and their WT littermates received 3% DSS in their drinking water to induce acute and chronic colitis, or untreated drinking water as a control. Multiple observations collectively indicated that gp130^757F/F^ mice were considerably more resistant to acute DSS-induced colitis than WT littermates. In WT mice, DSS treatment caused severe weight loss toward the end of the treatment. However, weight loss in gp130^757F/F^ mice was significantly attenuated (Fig. 1A). As shown in Fig. 1B, shortening of the colon, which is a macroscopic indication of colitis, was observed in DSS-treated WT mice but not acutely DSS-treated gp130^757F/F^ mice. Histological examination of colonic sections revealed disruption of the colonic mucosa with significant ulceration in WT mice, whereas gp130^757F/F^ mice retained intact architecture of the colonic mucosa (Fig. 1C,D). In chronic DSS-induced colitis, both, WT mice and gp130^757F/F^ mice, showed significant weight loss and shortening of the colon on day 44 after four repetitive cycles of DSS treatment (Fig. 2). However, the weight loss of gp130^757F/F^ mice was significantly attenuated during the first two cycles of DSS treatment (day 12–21) (Fig. 2A) and histological examination of colonic sections showed reduced disruption of the colonic mucosa with significant less ulcerations (data not shown). Importantly, weight loss attenuation in gp130^757F/F^ mice during chronic DSS-induced colitis occurred only through the first two cycles but not the latter two.

### Expression of cytokines is attenuated in gp130^757F/F^ mice with acute DSS-induced colitis

**Figure 3:**
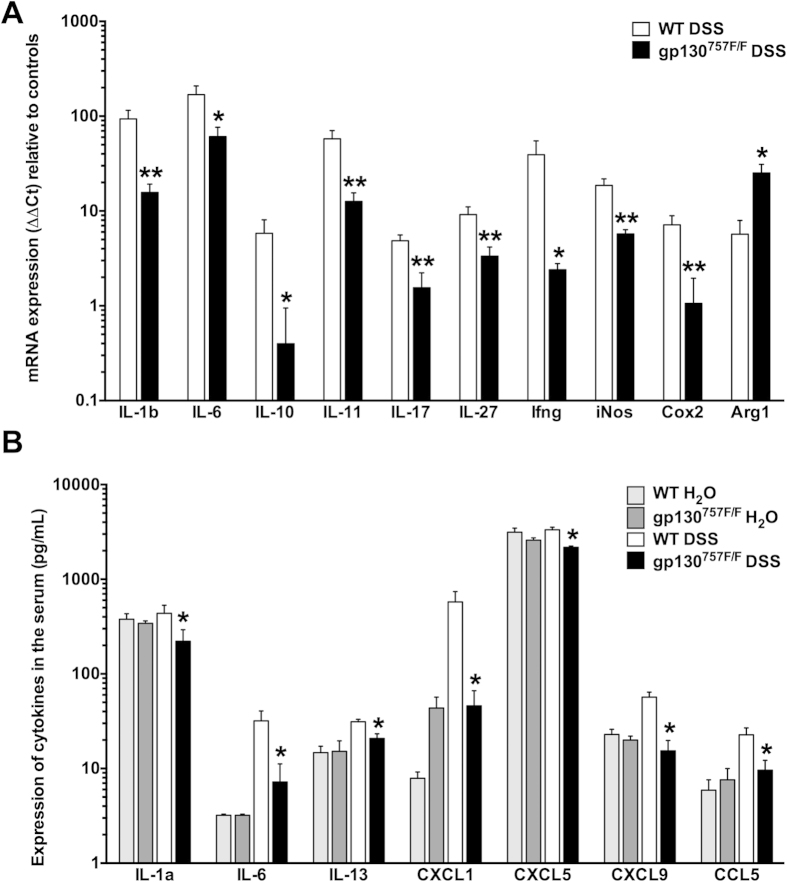
Cytokine expression in acute DSS-induced colitis. **(A)** mRNA was extracted from colons of DSS-treated wild type (WT, n = 16) and gp130^757F/F^ (n = 20) mice and subjected to qPCR analysis for a range of cytokines and inflammatory mediators. Expression of specific mRNA is standardized against RPL32 and expressed as fold change compared to untreated wild type mice (n = 6) by the ΔΔCt method. **(B)** Blood was collected on day 9 when mice were euthanized. Serum cytokines of untreated and DSS-treated wild type (WT) and gp130^757F/F^ mice were analysed by luminex array. Bars refer to mean ± SEM of three independent experiments. *indicates *P* < 0.05 and **indicates *P* < 0.01 in mice of different genotypes both receiving DSS.

In agreement with the observed protection of gp130^757F/F^ mice from acute DSS-induced colitis we found that gene expression of the pro-inflammatory mediators IL-1β, IL-6, IL-11, IL-17, IL-27, IFNγ and iNOS are all significantly reduced in the colons of DSS-treated gp130^757F/F^ mice (day 9) compared with DSS-treated WT littermates (Fig. 3A). Likewise, serum levels of cytokines and chemokines, including IL-1α, IL-6, CXCL1, CXCL5, CXCL9 and CCL5, were markedly lower in gp130^757F/F^ mice (day 9) than in WT mice following DSS treatment (Fig. 3B). Interestingly, gene expression of arginase 1 (ARG1) was significantly induced in colons of DSS-treated gp130^757F/F^ mice (25.17 ± 5.86 SEM; n = 5) compared with DSS-treated WT mice (5.69 ± 2.25 SEM; n = 5; P < 0.05).

IL-6 mRNA expression in the colon (Fig. 3A) correlated with the expression in the serum (Fig. 3B). The mRNA expression of IL-1β, IL-10, IL-17, and IFNγ was decreased in the colon of DSS-treated gp130^757F/F^ mice compared to DSS-treated WT mice, whereas we found no difference in the expression levels of these cytokines in sera of these mice. However, it has been reported before that serum concentrations of these cytokines (IL-1β, IL-10, IFNγ) are not significantly changed in acute DSS colitis^27^.

### gp130 signalling is not altered in the colon but STAT3 phosphorylation is enhanced in macrophages of gp130^757F/F^ mice

The gp130 receptor (β) binds the IL-6 family cytokines along with each of their specific transmembrane receptor α-subunits in a hexadimeric complex, followed by activation of receptor-associated JAKs. JAKs activate recruited STATs 1 and 3 by phosphorylation, which then translocate to the nucleus and initiate transcription of target genes involved in cell survival, proliferation and inflammation. The suppressor of cytokine signalling 3 (SOCS3) is up-regulated in response to STAT3 and can bind to a tyrosine residue at position Y757 of the gp130 receptor in mice and block signal transduction in a negative feedback-loop. gp130^757F/F^ mice with a phenylalanine (F) for tyrosine (Y) substitution at the 757 residue show sustained STAT3 signalling due to the lack of SOCS3 binding and reduced Src-homology tyrosine phosphatase 2 (SHP2) activity which also normally competes with SOCS3 for Y757 binding, and activates RAS/MAP kinase/ERK1/2 pathways as well as inhibiting STAT3 phosphorylation^19^,^20^.

**Figure 4:**
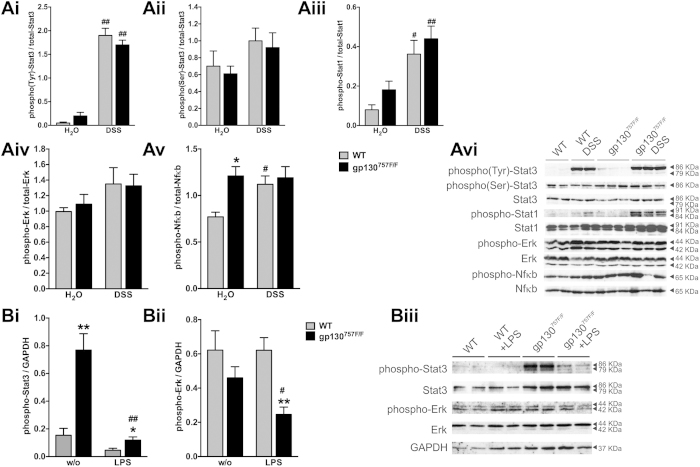
gp130 signalling in macrophages and colon of gp130^757F/F^ mice. **(A)** Acute colitis was induced in wild type (WT) mice and gp130^757F/F^ mice with 3% DSS in drinking water and compared with untreated mice (H_2_O). At least n = 5 mice were assessed in each group in three independent experiments. Proteins were extracted from removed colons on day 9 and quantified by Western blotting for total- and phospho (Tyr)-STAT3 (**Ai**), total- and phospho (Ser)-STAT3 (**Aii**), total- and phospho STAT1 (**Aiii**), total- and phospho ERK1/2 (**Aiv**) and total- and phospho NFκB (**Av**). Data is expressed as mean optical density (±SEM) of phosphorylated protein bands normalized to respective total protein bands. Representative blots for Ai-Av are shown (**Avi**). *indicates *P* < 0.05 compared to mice with different genotype; ^#^indicates *P* < 0.05 and ^##^indicates *P* < 0.01 compared to untreated mice with the same genotype. **(B)** Peritoneal macrophages were harvested from gp130^757F/F^ (n = 6) or wild type (WT) mice (n = 6) and treated *in vitro* with LPS (100 ng/mL) for 3 hours. Proteins were extracted from cultured macrophages and quantified by Western blotting for total- and phospho (Tyr)-STAT3 (**Bi**) and total- and phospho ERK1/2 (**Bii**). The intensity of the signal was quantified by densitometry and phosphorylated proteins expressed as a proportion of GAPDH from a duplicate membrane. Bars represent the mean ± SEM of three independent experiments. Representative blots for Bi and Bii are shown (**Biii**). *indicates *P* < 0.05 and **indicates *P* < 0.01 in macrophages of mice with different genotype; ^#^indicates *P* < 0.05 and ^##^indicates *P* < 0.01 compared to untreated macrophages of mice with the same genotype.

To investigate whether inhibition of colitis in gp130^757F/F^ mice is due to altered gp130 signalling, we examined activation of signalling molecules STAT1, STAT3, ERK1/2 and NfκB in the colon of DSS-treated and untreated gp130^757F/F^ and WT mice (Fig. 4). Interestingly, activation by phosphorylation of STAT3 (Tyr705 and Ser727), STAT1 (Tyr701) or ERK1/2 (Thr202/Tyr204) were not increased in the colon of gp130^757F/F^ compared with WT mice (Fig. 4Ai–iv). The basal NFκB (Ser276) activation was increased in the colon of gp130^757F/F^ mice compared with WT mice, but was not increased further following DSS treatment (Fig. 4Av). STAT1 and STAT3 activation in the colon was increased after DSS treatment, both, in gp130^757F/F^ and WT mice (Fig. 4Aii,iii). However, we found that activation of STAT3 is significantly increased in peritoneal macrophages from gp130^757F/F^ mice compared with WT mice (Fig. 4Bi). LPS-stimulated peritoneal macrophages of gp130^757F/F^ mice showed increased STAT3 and decreased ERK1/2 activation compared to WT mice (Fig. 4Bi,ii). Taken together, altered total colonic STAT3 activation does not account for resistance to acute DSS-induced colitis in gp130^757F/F^ mice, but the altered susceptibility may be due to the STAT3 activation in myeloid cells.

### LysMcre/STAT3^flox^ mice are not protected from acute and chronic DSS-induced colitis

**Figure 5:**
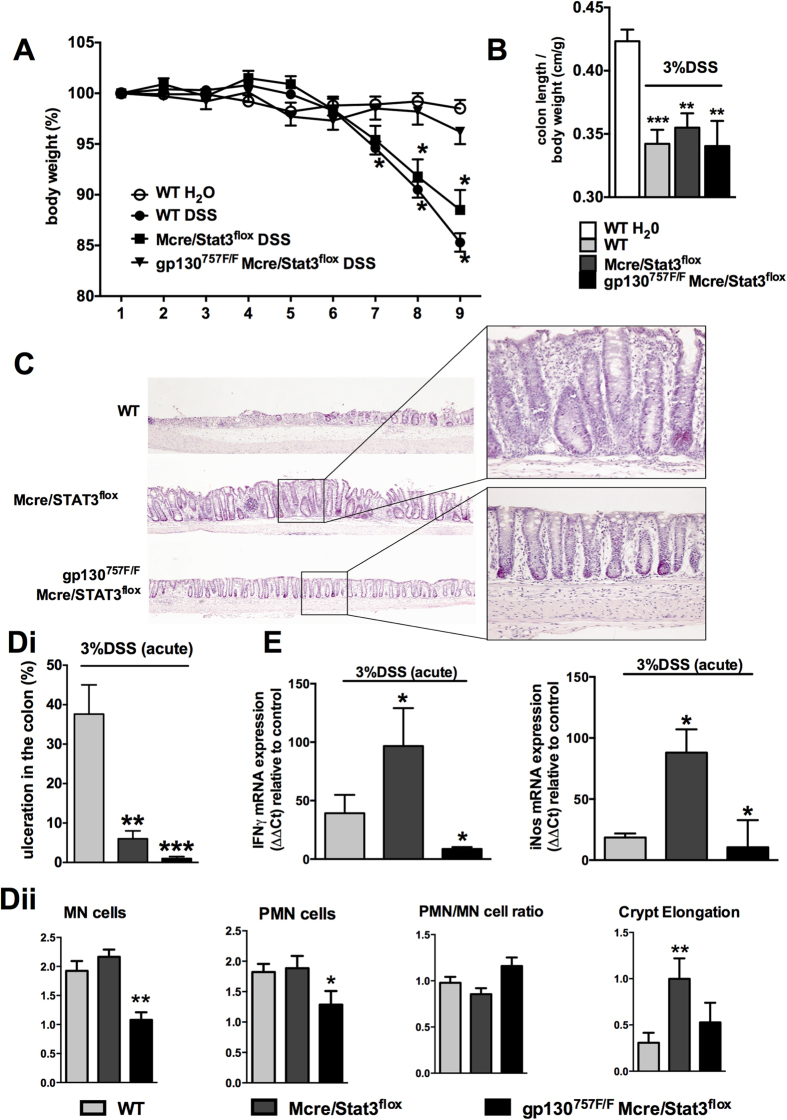
Susceptibility to acute colitis in LysMcre/STAT3^flox^ and gp130^757F/F^ LysMcre/STAT3^flox^ mice. **(A)** Acute colitis was induced in wild type (WT) mice (n = 15), LysMcre/STAT3^flox^ mice (n = 15) and gp130^757F/F^ LysMcre/STAT3^flox^ mice (n = 7) with 3% DSS in drinking water and compared with untreated mice (H_2_O, n = 16). The daily changes in body weight were calculated over the course of at least three independent experiments. *indicates *P* < 0.05 compared to mice receiving only water. **(B)** On day 9 of acute colitis colons were removed. The graph shows the mean colon length per body weight (±SEM) of each experimental group. **(C)** Microscopic analysis of H&E-stained colon sections was performed. Representative microscopic colon images of mice with colitis and healthy control mice are shown (H&E staining). **(Di)** The proportion of ulcerated colon relative to the whole colonic mucosa was calculated (acute colitis model). **(Dii)** Semi-quantitative histological comparative analysis of WT and gp130^757F/F^ mice was performed. **(E)** mRNA was extracted from colons (acute colitis model) and subjected to qPCR analysis for IFNγ and iNOS. Expression of specific mRNA is standardized against RPL32 and expressed as fold change compared to untreated wild type mice by the -2ΔΔCt method. *indicates *P* < 0.05; **indicates *P* < 0.01 and ***indicates *P* < 0.001 compared to control.

**Figure 6:**
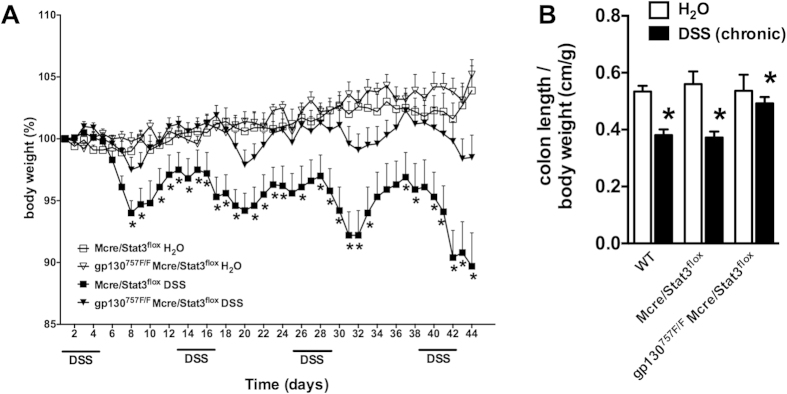
Susceptibility to chronic colitis in LysMcre/STAT3^flox^ and gp130^757F/F^ LysMcre/STAT3^flox^ mice. **(A)** Chronic colitis was induced in LysMcre/STAT3^flox^ mice (n = 23) and gp130^757F/F^ LysMcre/STAT3^flox^ mice (n = 24) with 3% DSS in drinking water on days indicated and compared with genotype-matched untreated mice (H_2_O, n = 20 in each group). The daily changes in body weight were calculated over the course of at least three independent experiments. *indicates *P* < 0.05 in mice of different genotype both receiving DSS. **(B)** The graph shows the mean colon length per body weight (±SEM) on day 44 of each experimental group (chronic colitis). *indicates *P* < 0.05 compared to control.

To explore whether STAT3 activation in myeloid cells accounts for resistance to acute DSS-induced colitis in gp130^757F/F^ mice, we analyzed the susceptibility to acute and chronic DSS-induced colitis in mice with myeloid-specific STAT3-deficiency (LysMcre/STAT3^flox^ mice and gp130^757F/F^ LysMcre/STAT3^flox^ mice) (Fig. 5). LysMcre/STAT3^flox^ mice were not protected from acute and chronic DSS-induced colitis and showed progressive and severe weight loss and shortening of the colon on day 9 (acute colitis model; Fig. 5A,B) and on day 44 (chronic colitis model; Fig. 6A,B), respectively. In contrast, we found that mice with myeloid-specific STAT3-deficiency but simultaneous systemic hyperactivation of STAT3 (gp130^757F/F^ LysMcre/STAT3^flox^ mice) were protected from acute and chronic DSS-induced colitis as indicated by the lack of weight loss (acute (Fig. 5A) and chronic colitis (Fig. 6A) model), no shortening of the colon on day 44 (chronic colitis model, Fig. 6B) and normal architecture of the colonic mucosa on day 9 (acute colitis model; Fig. 5C,D). Surprisingly, we found that protected gp130^757F/F^ LysMcre/STAT3^flox^ mice showed shortening of the colon (Fig. 5B) and that susceptible LysMcre/STAT3^flox^ mice had significantly less ulcerations of the colonic mucosa in the acute DSS-induced colitis model (Fig. 5Di). However, gp130^757F/F^ LysMcre/STAT3^flox^ mice with DSS-induced colitis showed significantly lower mononuclear and polymorphonuclear cell infiltrates in the colon compared to LysMcre/STAT3^flox^ and WT mice and LysMcre/STAT3^flox^ mice with colitis had a significantly higher crypt elongation score compared to gp130^757F/F^ LysMcre/STAT3^flox^ and WT mice. Furthermore, gene expression of IFNγ and iNOS, which play critical roles in mediating the inflammatory response during acute DSS colitis^28^,^29^, was significantly increased on day 9 in the colon of DSS-treated LysMcre/STAT3^flox^ mice but significantly decreased in the colon of gp130^757F/F^ LysMcre/STAT3^flox^ mice (Fig. 5E).

### Gene expression of cytokines IL-19 and IL-33 is markedly increased in gp130^757F/F^ mice

**Figure 7:**
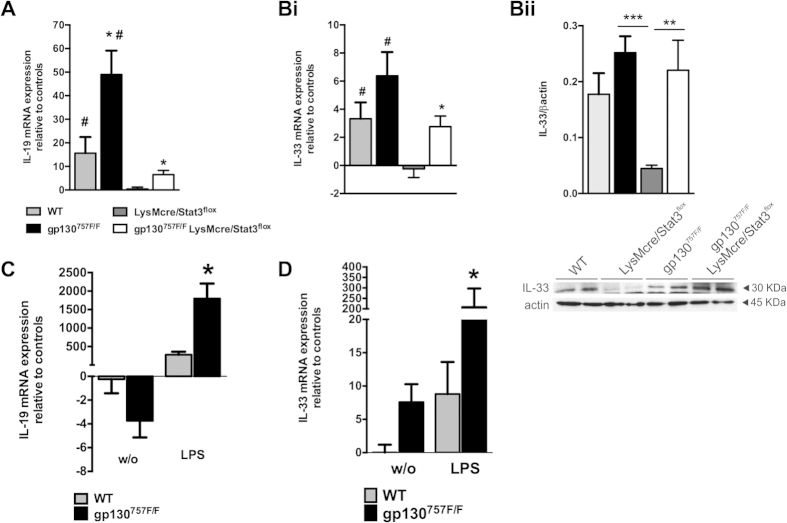
Consequences of altered gp130 signalling on IL-19 and IL-33 gene expression. **(A,B)** mRNA was extracted from colons of mice with acute DSS-induced colitis (as indicated; n = 20 per group) and untreated control mice (not shown) and subjected to qPCR analysis for IL-19 (**A**) and IL-33 (**Bi**) transcripts. *indicates *P* < 0.05 (compared to DSS-treated wild type); ^#^indicates *P* < 0.05 (compared to respective genotype-matched untreated control). (**Bii**) Protein extracts from DSS treated colons were quantified by Western blotting with antibodies to IL-33 and β-Actin. Data is expressed as mean optical density (±SEM) of IL-33 protein bands normalized to respective β-Actin protein bands. Representative blots for Bii are shown. **indicates *P* < 0.01 and ***indicates *P* < 0.001. **(C,D)** Peritoneal macrophages were harvested from gp130^757F/F^ or wild type (WT) mice (n = 6 per group) and treated *in vitro* with lipopolysaccharide (LPS, 100 ng/mL). mRNA was extracted and subjected to qPCR analysis for IL-19 (**C**) and IL-33 (**D**) transcripts. Bars refer to mean (±SEM) of 3 independent experiments. *indicates *P* < 0.05 (compared to wild type).

Our analyses of gene expression in WT, gp130^757F/F^, LysMcre/STAT3^flox^ and gp130^757F/F^ LysMcre/STAT3^flox^ mice on day 9 of acute DSS-induced colitis showed that the expression of IL-19 (Fig. 7A) and IL-33 (Fig. 7Bi) in the colon is i) significantly increased in gp130^757F/F^ mice; ii) suppressed in LysMcre/STAT3^flox^; and iii) unchanged in gp130^757F/F^ LysMcre/STAT3^flox^. To confirm that RNA expression of these cytokines in the colon of mice with acute DSS-induced colitis correlates with protein data we exemplarily provide protein levels of IL-33 (Fig. 7Bii). Protein levels of IL-33 (Fig. 7Bii) correlated with gene expression data (Fig. 7Bi). IL-19 and IL-33 are known to have beneficial roles during disease attenuation in different models of experimental colitis, including acute and chronic DSS-induced colitis^30^,^31^,^32^,^33^. Based on our findings we therefore hypothesized that the altered colitis susceptibility of gp130^757F/F^ mice may be due to STAT3 activation in myeloid cells, and determined the expression of IL-19 and IL-33 in peritoneal macrophages of WT and gp130^757F/F^ mice with and without LPS stimulation. The gene expression of IL-19 and IL-33 in LPS-stimulated peritoneal macrophages of gp130^757F/F^ mice was significantly increased compared with LPS-stimulated peritoneal macrophages of WT mice (Fig. 7C,D). The basal gene expression of IL-33 was also significantly increased in peritoneal macrophages of gp130^757F/F^ mice compared with WT mice (Fig. 7D). Thus, altered STAT3 signalling in gp130^757F/F^ mice may modulate colitis susceptibility in these mice via increased expression of protective/suppressive cytokines in myeloid cells.

Because of the crucial role of STAT3 in the expansion of myeloid-derived suppressor cells, we then analyzed the influence of altered gp130/STAT3 signalling on the frequency, phenotype and functional properties of myeloid cell populations during DSS-induced colitis. MDSC activation results in the upregulation of immunosuppressive factors, such as ARG1, and an increase in the production of anti-inflammatory Th2 cell cytokines. Because of the observed increase in the expression of ARG1 in the colon of gp130^757F/F^ mice (Fig. 3A), we next focused our attention on the role of MDSCs in decreased colitis susceptibility of gp130^757F/F^ mice.

### DSS treatment results in a marked increase in MDSCs and arginase activity in the colon of gp130^757F/F^ mice

**Figure 8:**
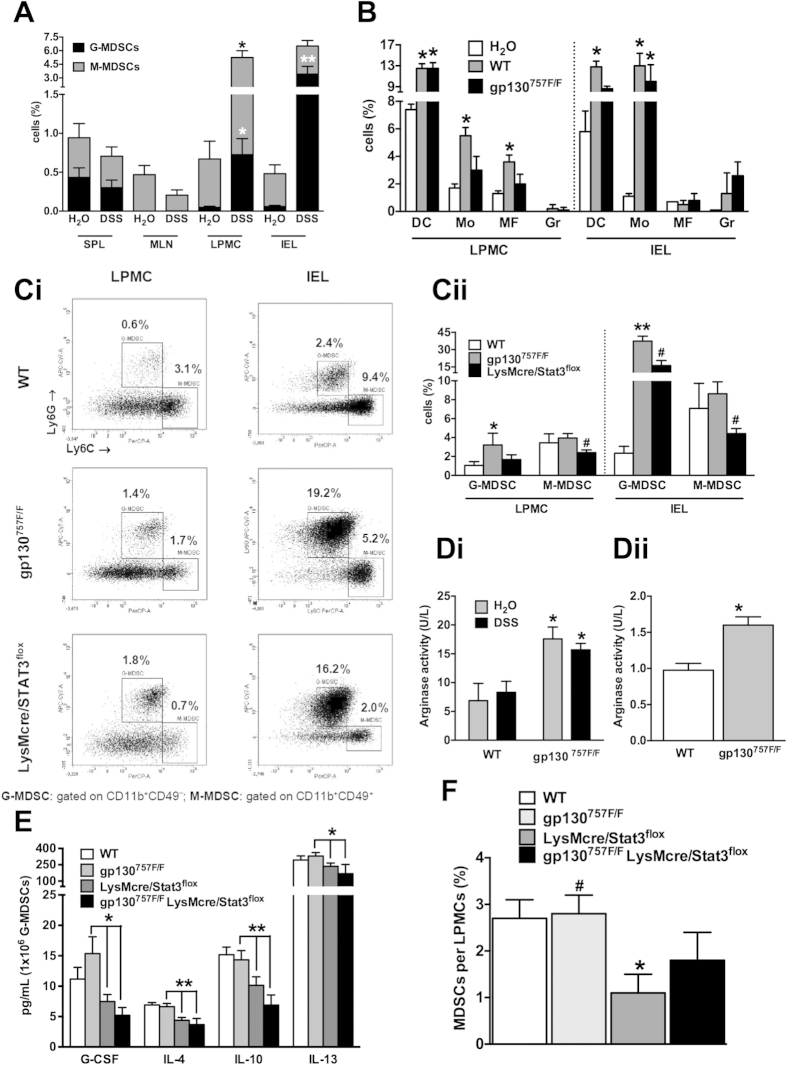
The effect of altered gp130 signalling on MDSCs during intestinal inflammation. Wild type (WT), gp130^757F/F^, LysMcre/STAT3^flox^ and gp130^757F/F^ LysMcre/STAT3^flox^ mice were treated with the acute (**A–E**) or chronic (**F**) DSS protocol to induce colitis and compared with untreated mice (H_2_O). **(A)** Cell populations from spleens (SPL), mesenteric lymph nodes (MLN), intraepithelial lymphocytes (IEL) and lamina propria mononuclear cells (LPMC) of wild type mice were characterized by flow cytometry for subpopulations of CD11b^+^Ly6C^low^Ly6G^+^CD49d^−^ granulocytic MDSCs (G-MDSC) and CD11b^+^Ly6C^high^Ly6G^−^CD49d^+^ monocytic MDSCs (M-MDSC). **(B)** Colonic cell population from IELs and LPMCs of untreated WT mice (H_2_O) and DSS-treated WT and gp130^757F/F^ mice were characterized by flow cytometry for subpopulations of CD11c^+^F4/80^−^Ly6G^−^Ly6C^−^ dendritic cells (DC), CD11c^−^CD11b^+^Ly6G^−^F4/80^+^ monocytes (Mo), CD11b^−^F4/80^+^Ly6C^−^Ly6G^−^ macrophages (MΦ) and CD11b^+^Ly6G^+^Ly6C^low^F4/80^−^CD11c^–^ granulocytes (Gr). **(C)** Colonic cell population from IELs and LPMCs of DSS-treated wild type (WT) and gp130^757F/F^ mice were characterized by flow cytometry for subpopulations of CD11b^+^Ly6C^low^Ly6G^+^CD49d^−^ granulocytic MDSCs (G-MDSC) and CD11b^+^Ly6C^high^Ly6G^−^CD49d^+^monocytic MDSCs (M-MDSC). **(D)** Arginase activity was determined in homogenized colon tissue of DSS-treated or untreated (H_2_O) wild type (WT) and gp130^757F/F^ mice (**Di**) and also in polymorphonuclear Gr1^high^Ly6G^+^ MDSCs isolated from spleens of wild type (WT) and gp130^757F/F^ mice (**Dii**). **(E)** Granulocytic Gr1^high^Ly6G^+^ MDSCs (G-MDSCs) were isolated from spleens of wild type (WT), gp130^757F/F^, LysMcre/STAT3^flox^ and gp130^757F/F^ LysMcre/STAT3^flox^ mice and 1 × 10^6^ cells were cultured for 24 hours. Cell culture supernatants were analysed by luminex array. **(F)** Cell populations from lamina propria mononuclear cells (LPMCs) were isolated from the colon of mice with chronic DSS-induced colitis and characterized by flow cytometry for MDSC subpopulations (CD11b^+^CD45^+^CD11c^-/int^, Gr1^high/low^). *indicates *P* < 0.05 and **indicates *P* < 0.01 (compared to untreated control or wild type); ^#^indicates *P* < 0.05 (compared to LysMcre/STAT3^flox^ or gp130^757F/F^ mice). Bars represent the mean±SEM of at least three independent experiments (n = 5–12 per group).

First, we induced acute DSS-colitis in WT mice and determined the frequency and phenotype of granulocytic (G) and monocytic (M) MDSCs in cell populations from the spleen, mesenteric lymph nodes (MLN), and colonic lamina propria mononuclear cells (LPMCs) and intraepithelial lymphocytes (IELs), using flow cytometry. The frequency of G-MDSCs and M-MDSCs did not change in spleen or MLN following DSS treatment compared with water-treated control mice. In contrast, DSS treatment resulted in a marked increase in the number of M-MDSCs and G-MDSCs in LPMCs and a marked increase in the number of G-MDSCs in IELs (Fig. 8A). We next compared the frequency of other myeloid cells including dendritic cells (DCs), monocytes (Mo), macrophages (MΦ) and granulocytes (Gr) in the colon (LPMCs and IELs) of DSS-treated or untreated WT and gp130^757F/F^ mice (acute colitis model). The frequency of these cell types was not significantly different in the colon of gp130^757F/F^ mice following DSS treatment when compared to WT mice (Fig. 8B). In contrast, the frequency of G-MDSCs (but not M-MDSCs) was significantly increased in LPMCs and IELs of DSS-treated gp130^757F/F^ mice when compared to WT mice (Fig. 8Cii). Furthermore, the frequency of G- and M-MDSCs in the colon after DSS treatment was significantly reduced in LysMcre/STAT3^flox^ with myeloid-specific STAT3-deficiency compared with gp130^757F/F^ mice with hyperactivation of STAT3 (Fig. 8C).

The STAT3 mRNA expression (fold change) in MDSCs isolated from spleens was significantly increased in gp130^757F/F^ mice (3.10 ± 1.12 SEM) compared to WT mice (0.18 ± 0.09 SEM; *P *= 0.05) and LysMcre/Stat3^flox^ mice (-0.33 ± 1.06 SEM; *P *= 0.05). In addition, recent findings suggest that STAT3 also regulates MDSC expansion by inducing the expression of S100 calcium-binding protein A8 (S100A8) and S100A9, the receptors for which are also expressed on the cell surface of MDSCs^4^. Therefore, we analyzed the STAT3-driven expression of the MDSC markers S100A8 and S100A9 as a functional measure of STAT3 activation. Gene expression of S100A8 (6.00 ± 2.40 SEM) and S100A9 (1.98 ± 0.01 SEM) in MDSCs isolated from spleens of gp130^757F/F^ mice was significantly increased compared to WT mice (*P *= 0.05) indicating a STAT3 activation in MDSCs in gp130^757F/F^ mice.

Based on our finding that gp130^757F/F^ mice have increased numbers of G-MDSCs and gene expression of ARG1 in colons following DSS treatment (Fig. 3A), we next analyzed the arginase activity in colons of DSS-treated and untreated WT and gp130^757F/F^ mice. gp130^757F/F^ mice showed an increased basal arginase activity in colons compared with WT mice, which was not increased further following DSS treatment (Fig. 8Di). Likewise, G-MDSCs isolated from spleens of gp130^757F/F^ mice showed a higher basal arginase activity than splenic G-MDSCs from WT mice (Fig. 8Dii). Furthermore, splenic G-MDSCs of gp130^757F/F^ mice produced significantly higher levels of Th2 cell cytokines (IL-4, IL-10 and IL-13) and G-CSF compared to mice with myeloid-specific STAT3-deficiency (Fig. 8E). Finally, we could show that in the model of chronic DSS-induced colitis, MDSC subpopulations in the colon were significantly reduced in LysMcre/STAT3^flox^ mice compared to WT mice, whereas the frequency of MDSCs was significantly higher in gp130^757F/F^ mice, and not significantly altered in gp130^757F/F^ LysMcre/STAT3^flox^ mice (Fig. 8F).

## Discussion

In this study, we demonstrate that the resistance to chemically induced colitis in mice with altered gp130 signalling is via myeloid-cell specific STAT3 activation, subsequent expansion of granulocytic MDSC in the colon and increased production of suppressive and protective mediators.

Firstly, we show that gp130^757F/F^ mice are protected from acute DSS colitis. We found an increased expression and activity of ARG1 and a decreased expression of iNOS in colons of DSS-treated gp130^757F/F^ mice. The enzymes ARG1 and iNOS compete for L-arginine (L-Arg) as a substrate and the L-Arg metabolism in myeloid cells is a crucial component of T cell suppression pathways^34^. iNOS produced by bone marrow-derived cells plays a critical role in mediating the inflammatory response during acute DSS- and 2,4,6-trinitrobenzene sulfonic acid (TNBS)-induced colitis^28^. iNOS deletion in *iNos*^−/−^ mice ameliorates colitis in the *Citrobacter rodentium* model and attenuates onset and severity of acute DSS-induced colitis^35^,^36^. ARG1 has a protective role in murine colitis by competitive inhibition of iNOS^36^. ARG1 activity is also required for alternatively activated M2 macrophage-mediated protection during DSS-induced colitis^37^. In addition, increased ARG1 levels are responsible for the suppressive mechanisms mediated by different subsets of MDSCs, which have been described in animal models of experimental colitis and human IBD^11^. The G-MDSC subset in particular is characterized by high ARG1 activity, low iNOS activity with low nitric oxide (NO) production and increased activity of STAT3^4^. STAT3 activation was not increased in the colon of gp130^757F/F^ mice but markedly increased in myeloid cells (peritoneal macrophages). We were therefore interested in the frequency, phenotype and functional properties of MDSCs in the colon of protected gp130^757F/F^ mice. We show that DSS treatment of WT mice does not lead do an increase in the frequency of G-MDSCs (with a CD11b^+^Ly6G^+^Ly6C^low^CD49^–^ phenotype) or M-MDSCs (with a CD11b^+^Ly6G^–^Ly6C^high^CD49^+^ phenotype) in spleen or MLN; whereas we noted a significant but modest increase of MDSC numbers in the colon during DSS-induced colitis. Previous studies on the frequency of MDSCs (with a CD11b^+^Gr1^+^ phenotype) in WT mice with DSS-induced colitis have reported divergent results. Haile *et al.* showed that DSS treatment of WT mice has no effect on the frequency of CD11b^+^Gr1^+^ cells in spleen or MLN^13^, whereas other studies showed that DSS treatment of WT mice results in an increase in CD11b^+^Gr1^+^ cells in the spleen and Peyer’s patches^16^,^38^,^39^. However, these studies confirmed that DSS treatment of WT mice had only a minimal effect on the frequency of CD11b^+^Gr1^+^ cells in colon^16^,^38^,^39^. Importantly, we show that the expansion of G-MDSCs in the colon (but not in spleen or MLN) of gp130^757F/F^ mice was markedly increased during acute DSS-induced colitis, and G-MDSCs of gp130^757F/F^ mice had a higher ARG1 activity than WT mice. In agreement with our data, it has been shown that systemic or myeloid cell-specific deletion of gp130 leads to a significant reduction of CD11b^+^Ly6G^+^ cell numbers in the colon of DSS-treated mice^40^. Contrariwise, it has been shown that an increased STAT3 activity is associated with an expansion of bone marrow CD11b^+^Ly6G^+^ cells, which in turn decreases the infiltration of neutrophils, reduces the level of serum IL-17 and ameliorates DSS-induced colitis^15^. It has also been noted previously, that that the majority of MDSCs present at the sites of colitis, express ARG1 while expressing minimal levels of iNOS^41^. Thus, it is conceivable that the protection of gp130^757F/F^ mice from acute DSS-induced colitis is via myeloid-specific STAT3 activation and expansion of immunosuppressive ARG1-expressing G-MDSC subsets in the colon. In this context it is important to note, that the adoptive/intravenous transfer of MDSCs in experimental models of colitis (DSS, TNBS, *IL-10*^−/−^, CD4^+^CD25^−^ T cell transfer into *Rag1*^−/−^ mice) is associated with amelioration of intestinal inflammation^11^,^13^,^14^,^15^,^16^,^17^,^38^,^39^,^41^, whereas anti-Gr-1 antibody treatment worsened DSS-induced colitis^38^. In addition to the gp130/STAT3-mediated effects on the expansion of mucosal MDSCs in DSS-induced colitis, we show that protected gp130^757F/F^ mice have increased gene expression levels of IL-19 in LPS-stimulated peritoneal macrophages and in the colon following DSS-treatment. Gene expression in the colon and serum levels of pro-inflammatory cytokines typically induced in DSS colitis was attenuated in gp130^757F/F^ mice with acute DSS colitis. It has been previously demonstrated that IL-19 regulates colonic inflammation and that acute DSS-induced colitis is exacerbated in *IL-19*^−/−^ mice^30^. IL-19-deficient macrophages produce significantly higher levels of pro-inflammatory cytokines such as IL-1β, IL-6, IL-12 and TNFα and show reduced levels of phosphorylation of STAT3. Thus, the gp130/STAT3-mediated expression of IL-19 in our study could further contribute to the protection of gp130^757F/F^ mice from acute DSS-induced colitis. However, the exact role of IL-19 in intestinal inflammation is beyond the scope of the present study and remains to be established in future studies.

Secondly, we show that LysMcre/STAT3^flox^ mice with myeloid-specific STAT3-deficiency are not protected from acute and chronic colitis. It has previously been shown that STAT3-deficiency in macrophages and neutrophils leads to chronic T cell mediated colitis secondary to the inability of myeloid cells to respond to IL-10^21^,^22^. These data indicate that targeted STAT3 deletion in MDSCs leads to uncontrolled T cell activation and initiates intestinal inflammation. Here, we show that the expansion of G-MDSCs in the colon of LysMcre/STAT3^flox^ mice is reduced during acute and chronic DSS-induced colitis and that the secretion of anti-inflammatory Th2 cell cytokines (IL-4, IL-10, IL-13) by MDSCs of LysMcre/STAT3^flox^ mice is suppressed. These findings support the idea that the protection of gp130^757F/F^ mice from acute DSS-induced colitis is via myeloid-specific STAT3 activation and expansion of immunosuppressive G-MDSC subsets in the colon. Furthermore, in contrast to gp130^757F/F^ mice and even WT mice, gene expression of iNOS and IFNγ was increased and gene expression of IL-19 and IL-33 was abrogated in the colon of LysMcre/STAT3^flox^ mice with acute DSS-induced colitis. IFNγ is known to be causatively involved in acute DSS colitis, whereas the induction of iNOS seems to act as a critical toxic effector molecule in the pathogenesis of chronic DSS-induced colitis^29^,^42^. IL-19 has, in addition to its protective role in experimental colitis (as mentioned above), a potential anti-inflammatory role in human IBD^43^,^44^. IL-19 is strongly up regulated in the presence of Th2-type cytokines (IL-4, IL-13)^45^. Thus, the abrogated IL-19 expression in the colon of DSS-treated LysMcre/STAT3^flox^ mice could be related to our observation that in LysMcre/STAT3^flox^ mice i) G-MDSCs secrete significantly lower levels of the Th2 cytokines IL-4, IL-10 and IL-13; and ii) G-MDSC numbers are reduced in the colon during DSS-induced acute colitis. Finally, IL-33 is known to have extenuating effects in chronic DSS-induced colitis by shifting the immune response towards a Th2-like reaction^31^. Interestingly, IL-33 treatment of mice with chronic DSS-induced colitis leads to an increase in the number of CD11b^+^Ly6G^+^ (but not CD11b^+^Ly6G^−^) cells in the lamina propria. Other studies showed that IL-33 ameliorates the development of experimental colitis by a Th1-to-Th2 switch and expansion of regulatory T cells and via expansion of IL-10-producing regulatory B cells^32^,^33^. However, the exact role of IL-33 in intestinal inflammation is beyond the scope of the present study and remains to be established in future studies^46^.

Thirdly, we show that gp130^757F/F^ LysMcre/STAT3^flox^ mice are protected from acute and chronic colitis and that gp130^757F/F^ mice are not completely protected from chronic DSS colitis. The number of MDSCs in the lamina propria of gp130^757F/F^ LysMcre/STAT3^flox^ and gp130^757F/F^ mice with chronic DSS-induced colitis was not increased compared with WT mice. Likewise, gene expression of the protective cytokines IL-19 and IL-33 in the colon of gp130^757F/F^ LysMcre/STAT3^flox^ mice during acute DSS-induced colitis was similar to the gene expression levels in WT mice. Thus, other factors must play a role in the protection from colitis in gp130^757F/F^ LysMcre/STAT3^flox^ mice. We show that the gene expression of IFNγ and iNOS was attenuated in the colon of gp130^757F/F^ LysMcre/STAT3^flox^ mice with acute DSS-induced colitis. Both mediators play a pathogenic role in the development of DSS-induced colitis as discussed above^29^,^42^.

Future studies will be needed to investigate the T cell suppressive mechanisms and the tissue-specific effects of MDSC from gp130^757F/F^ mice in more detail. In this regard, the adoptive transfer of MDSCs from gp130^757F/F^ mice into *Rag1*^−/−^ mice (which do not develop functional T cells) with DSS- or CD4^+^CD25^−^ T cell transfer-induced colitis could help to reveal the underlying contribution of innate and adaptive immune responses^47^. In addition, an interesting future question will be whether MDSCs from gp130^757F/F^ mice are able to induce regulatory T cells (Treg). Zhang *et al.* showed that depletion of Gr1^+^CD11b^+^ cells of MDSC character by anti-GR-1 treatment exacerbated DSS-induced colitis from the findings of body weight loss, colon length and disease activity index^38^. However, anti-GR-1 treatment is not exclusively MDSC-specific and it is technically extremely difficult to specifically deplete MDSCs. Finally, it will be important in the future to translate our understanding of MDSC functions during murine colitis and to characterize the role of MDSCs in human IBD. Given the multifaceted function of MDSCs in the amelioration of IBD models associated with the suppression or induction of specific types of the immune response, MDSCs not only have immunosuppressive functions but could also belong to the network of myeloid regulatory cells with immunoregulatory functions^48^. However, these questions are beyond the scope of our present study and will be left for future investigations.

**Figure 9:**
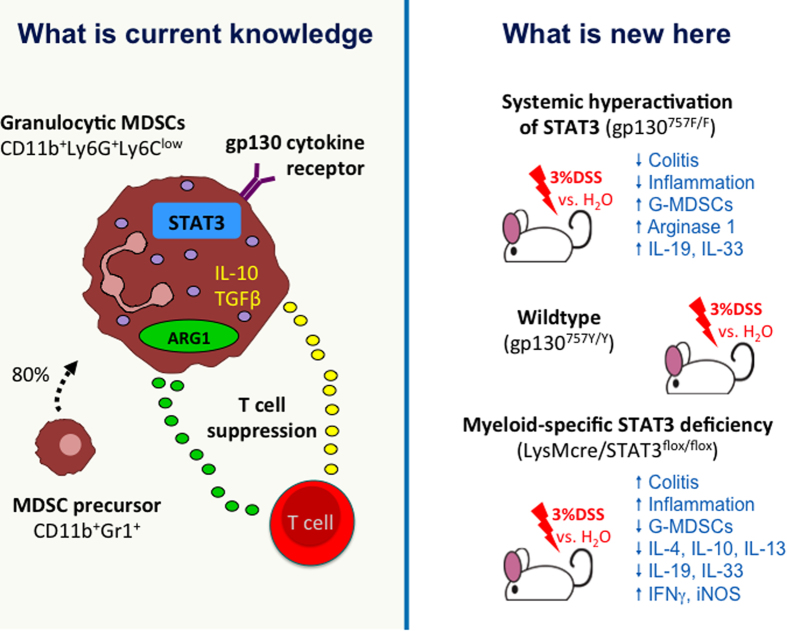
gp130/STAT3 signaling and MDSCs in colitis. **WHAT IS CURRENT KNOWLEDGE** Myeloid derived suppressor cells (MDSCs) are a heterogeneous group of immature cells that includes precursors of macrophages, granulocytes, dendritic cells and myeloid cells at earlier stages of differentiation. They are defined by their co-expression of Gr-1 and CD11b. MDSCs regulate immune responses and tissue repair in healthy individuals and MDSCs rapidly expands during inflammation, infection and cancer. It is predominantly (70-90%) the granulocytic subset of MDSCs (G-MDSC) that expands, which has a CD11b^+^Ly6G^+^Ly6C^low^ phenotype. G-MDSCs have increased activity of signal transducer and activator of transcription 3 (STAT3), which is activated by binding of cytokines to the glycoprotein (gp)130 receptor and regulates the expansion and survival of G-MDSC subsets. Activation of G-MDSCs leads to the upregulation of arginase 1 (ARG1), which causes the suppression of T cell responses, and to the increased production of other suppressive cytokines, such as interleukin (IL-)10 and transforming growth factor-β (TGF-β). **WHAT IS NEW HERE** gp130^757F/F^ mice with a phenylalanine (F) for tyrosine (Y) substitution at the 757 residue of the gp130 receptor show sustained STAT3 signalling due to the inhibition of negative feedback loops, which normally activate alternative signalling pathways downstream of gp130. Experimental colitis, induced with 3% dextran sulphate sodium (DSS) in drinking water in gp130^757F/F^ mice and compared to water (H_2_O)-treated gp130^757F/F^ and wildtype (gp130^757Y/Y^) mice, resulted in reduced disease severity and amelioration of intestinal inflammation with expansion of G-MDSCs in the colon, increased arginase 1 expression and activity and increased expression of the protective cytokines IL-19 and IL-33 in the colon. LysMcre/Stat3^flox^ mice with myeloid-specific STAT3 deficiency, were not protected from DSS-induced colitis and colons showed reduced numbers of G-MDSCs, increased gene expression of pro-inflammatory interferon γ (IFNγ) and inducible nitric oxide synthase (iNos), and decreased expression of IL-19 and IL-33. Additionally, G-MDSCs of LysMcre/Stat3^flox^ mice produced significantly less anti-inflammatory cytokines, such as IL-4, IL-10 and IL-13, compared to gp130^757Y/Y^ mice. We suggest that the resistance to DSS-induced colitis in gp130^757F/F^ mice is via myeloid-cell specific STAT3 activation, MDSC expansion in the colon and increased production of suppressive and protective cytokines.

In summary, we suggest that the protective role of gp130-dependent STAT3 activation in experimental IBD involves the expansion and activation of mucosal MDSCs that express high levels of arginase 1 and anti-inflammatory Th2 cell cytokines (Fig. 9). Our data indicate that this gp130/STAT3-mediated immunoprotection in DSS-induced colitis is independent of the previously reported beneficial effects of STAT3 activation on intestinal epithelial cells in animal models of colitis-associated tumorigenesis. Thus, MDSCs might become a promising novel cell-based therapeutic option for patients with IBD.

## Materials and Methods

### Mice

gp130^757F/F^, LysMcre/STAT3^flox^ and gp130^757F/F^ LysMcre/STAT3^flox^ mice were used. gp130^757F/F^ mice harbor a phenylalanine (F) for tyrosine (Y) residue substitution at position 757 on the gp130 receptor, and show sustained STAT3 activation and signaling^23^. LysMcre/STAT3^flox^ conditional knockout mice are STAT3-deficient specifically in neutrophils and macrophages^22^. Compound gp130^757F/F^ LysMcre/STAT3^flox^ mice were generated from gp130^757F/F^ and LysMcre/STAT3^flox^ mice. All mice were backcrossed for a minimum of 8 generations onto a C57Bl/6 background and age matched wild type littermates were used as controls. Mice were housed under specific pathogen-free conditions in the animal facility of the Murdoch Children’s Research Institute. Mice were genotyped by multiplex PCR as previously described^49^. All animal experiments were performed with the approval of the Murdoch Children’s Research Institute Animal Ethics committee (AEC A713). All experiments on animals were performed in accordance with relevant and approved guidelines and regulations.

### Isolation of peritoneal macrophages

Peritoneal macrophages were recovered from mice from 5 mL HBSS injected into the peritoneal cavity. To enrich for macrophages, cells were resuspended in RPMI with 10% FCS, and plated onto uncoated plastic. Following a 10 minute incubation, non-adherent cells were removed and plated onto 6 well dishes. Adherent macrophage enriched cells were incubated for 16 hours in RPMI with 10% FCS, stimulated with LPS (100 ng/mL) and harvested 3 hours later. RNA was purified using the RNeasy mini kit according to the manufacturer’s instructions (Qiagen, Hilden, Germany).

### DSS-induced colitis

Both acute and chronic models of DSS-induced colitis were used. On days 1–6 gp130^757F/F^, LysMcre/STAT3^flox^ and gp130^757F/F^ LysMcre/STAT3^flox^ mice and matched wild type mice were provided with 3% DSS (dextran sulphate sodium; MW ~40 000; TdB Consultancy, Uppsala, Sweden) in drinking water ad libitum, or plain drinking water for controls. On day 7 all mice were provided standard drinking water, and then culled on day 9 (acute colitis). Alternatively, all mice were provided standard drinking water on days 6–12, and the protocol was repeated 4 times with mice culled on day 44, which was 3 days after the fourth DSS treatment when weight loss was the greatest prior to recovery (chronic colitis). All mice were weighed daily.

### Tissue preparation

Blood was collected by cardiac puncture, allowed to clot and serum isolated. Colons were removed from mice from cecum to the rectum and photographed with a Coolpix 4500 digital camera (Nikon Instruments, Melville, NY). Length of colon was measured from images using ImageJ software for Windows version 1.3 (NIH, Bethesda, MD)^50^. Colons were then either used for histological and molecular analysis or for flow cytometry (see below). For histological and molecular analysis mouse colon was bisected; one half was frozen and the other half fixed in 4% paraformaldehyde in PBS. The frozen tissue was either homogenized in PBS for enzyme assays or alternatively homogenized in Trizol (Invitrogen, Carlsbad, CA) and RNA, DNA and protein extracted according to the manufacturer’s instructions.

### Histological assessment and microscopic morphometry

Paraffin sections (4 μm) were stained with H&E. Images were captured with a Coolpix 4500 digital camera (Nikon Instruments, Melville, NY), and morphometric analysis was performed using ImageJ software for Windows version 1.3 (NIH, Bethesda, MD)^50^. For semi-quantitative histological analysis sections of the entire length of colon were scored for the following; 1) mononuclear cell (MN) infiltrate; 2) polymorphonuclear (PMN) cell infiltrate; and 3) crypt elongation according to the criteria; 0 none, 1 mild, 2 moderate, 3 severe. The PMN/MN ratio provides a simple and objective way of quantifying the degree of acute inflammation in clinical histopathology. For damage measurements, images of the entire colonic mucosa were captured and a digital line was drawn (and measured) along the muscularis mucosa in all regions where the epithelium was no longer intact (termed damaged). Length of damage was expressed relative to the total length of the mucosa.

### Isolation of intraepithelial lymphocytes and lamina propria mononuclear cells

Lamina propria tissue of colons was dissociated to single-cell suspensions by combining mechanical dissociation with enzymatic degradation of the extracellular adhesion proteins using a gentleMACS dissociator and a mouse lamina propria dissociation kit according to the manufacturer’s instructions (Miltenyi Biotec, Bergisch Gladbach, Germany). Initially, the intraepithelial lymphocytes (IELs) were disrupted from the mucosa by shaking the tissue in a predigestion solution. Then, the lamina propria tissue was further treated enzymatically and mechanically dissociated into a single-cell suspension containing lamina propria mononuclear cells (LPMCs) by using a gentleMACS dissociator. The resultant cell suspensions containing the LPMCs and IELs were further purified from contaminating epithelium, stroma and dead cells by centrifugation (1,000 × G, 45 minutes, 20 °C) on a 40/80% Percoll gradient (Sigma-Aldrich, St. Louis, MO).

### Isolation of splenocytes

Spleens were dissociated into single-cell suspensions by combining mechanical dissociation with enzymatic degradation of the extracellular matrix using a gentleMACS dissociator and a mouse spleen dissociation kit according to the manufacturer’s instructions (Miltenyi Biotec, Bergisch Gladbach, Germany). After dissociation, samples were applied to a 30 μm filter (Miltenyi Biotec, Bergisch Gladbach, Germany) to remove any remaining larger particles from the single-cell suspension.

### Isolation of mesenteric lymph nodes

Mesenteric lymph nodes (MLNs) were dissected from the mesentery and placed in HBSS containing 2 mM EDTA and 2% FBS on ice. MLNs were passed through a 70 μm cell strainer (BD Biosciences, Franklin Lakes, NJ) and washed twice with HBSS containing 2 mM EDTA and 2% FBS. Cell suspensions were centrifuged at 800 × G for 4 minutes at 4 °C and cells were resuspended for subsequent analysis.

### Flow cytometry

Purified cell populations from spleens, colons and mesenteric lymph nodes were stained with a cocktail of leucocyte cell surface markers including the following anti-mouse monoclonal antibodies: CD11b (Brilliant Violet 421 #101235 [1:1000]; PE #557397 [1:500]), CD49d (FITC #103605 [1:1000]), Gr1 (PerCP-Cy5.5 #552093 [1:1000]), Ly6C (PerCP #128028 [1:1000]), F4/80 (Alexa Fluor 700 #123130 [1:1000]), Ly6G (APC/Cy7 #127624 [1:500]), CD45 (V500 #561487 [1:500]) (all Biolegend, San Diego, CA) and CD11c (APC #17-0114-82 [1:1000]) (eBioscience, San Diego, CA). CountBright absolute counting beads (Molecular Probes, Eugene, OR) were used to determine total cell number per sample, and propidium iodide was used for viability. Data collection was performed on a BD LSR II Flow Cytometer and data analysis performed using FACSDiva (both BD Biosciences, Franklin Lakes, NJ). On the basis of forward and side scatter, propidium iodide staining, and staining on unutilized wave lengths, the following events were eliminated: debris, red blood cells, aggregates, dead cells and autofluorescence.

### Isolation of spleen-derived MDSCs

Splenocytes were isolated as described above. Granulocytic MDSCs were isolated by indirect magnetic labelling of Gr1^high^Ly6G^+^ cells with Anti-Ly6G-Biotin and Anti-Biotin MicroBeads using a mouse myeloid-derived suppressor cell kit (Miltenyi Biotec, Bergisch Gladbach, Germany) and subsequent magnetic separation using LS columns and a MidiMACS and QuadroMACS separator (all Miltenyi Biotec, Bergisch Gladbach, Germany). To increase the purity, the positively selected cell fraction containing the Gr1^high^Ly6G^+^ myeloid cells was separated over a second LS column. Isolated MDSCs were cultured for 24 hours in RPMI 1640 Glutamax medium supplemented with 10% fetal bovine serum (FBS), 2 mM non-essential amino acids, 50 IU penicillin and 50 μg/ml streptomycin (Invitrogen, Carlsbad, CA) at 37 °C in a humidified incubator with 5% CO_2_/air. Cell numbers of splenocytes and MDSCs were determined using the Moxi Z automated cell counter and type S cassettes (Orflo Technologies, Hailey, ID).

### Analysis of cytokines

Cytokine concentrations in serum and cell culture supernatants were analysed by Luminex array according to the manufacturer’s instructions. Briefly 50 μl of serum was analysed using MILLIPLEX multi-analyte panel mouse cytokine/chemokine magnetic bead panel (premixed 32-plex) immunology multiplex assay (Merck Millipore, Billerica, MA). This assay can detect the presence of the following cytokines/chemokines in sera: VEGF, Eotaxin, G-CSF, GM-CSF, IFN-γ, IL-1α, IL-1β, IL-2, IL-3, IL-4, IL-5, IL-6, IL-7, IL-9, IL-10, IL-12 (p40), IL-12 (p70), IL-13, IL-15, IL-17, IP-10, KC-like, LIF, LIX, MCP-1, M-CSF, MIG, MIP-1α, MIP-1β, MIP-2, RANTES, TNF-α. Likewise, 50 μl of cell culture supernatant was analysed using BIO-PLEX multi-analyte panel mouse cytokine/chemokine magnetic bead panel (premixed 23-plex) immunology multiplex assay (BIO-RAD, Hercules, CA), the Luminex 200 system with xPONENT 3.1 software (Luminex, Austin, TX). This assay can detect the presence of the following cytokines/chemokines:, Eotaxin, G-CSF, GM-CSF, IFN-γ, IL-1α, IL-1β, IL-2, IL-3, IL-4, IL-5, IL-6, IL-9, IL-10, IL-12 (p40), IL-12 (p70), IL-13, IL-17A, KC, MCP-1, MIP-1α, MIP-1β, RANTES and TNF-α.

### Quantitative real-time PCR

Total RNA was extracted using TRIzol reagent (Life Technologies, Carlsbad, CA). RNA (3 μg) was reverse transcribed into cDNA using Moloney murine leukemia virus reverse transcriptase primed with oligo(dT) 15 primer (Promega, Madison, WI). Quantitative PCR (qPCR) primers were designed using Primer Express software version 3.0.1 (Applied Biosystems, Foster City, CA). SYBR green chemistry (Promega, Madison, WI) was used with RPL32 as the internal reference gene. PCRs were performed and measured on a 7500 fast real-time PCR system and software version 2.0.6 (Applied Biosystems, Foster City, CA). Thermocycler conditions were 95 °C for 10 minutes, 40 cycles of 95 °C for 15 seconds and 60 °C for 15 seconds. Results were analyzed using sequence detector software, and relative fold differences were determined using the -2ΔΔCt method. Primer sequences for qPCR are given in Table 1.

### Immunoblotting

Protein extracts were prepared with TRIzol reagent (Life Technologies, Carlsbad, CA) according to the manufacturer’s instructions and 20 μg of extracts were subjected to sodium dodecyl sulphate/polyacrylamide gel electrophoresis. Membranes were blocked and incubated at 4 °C overnight in skim milk with the following antibodies: STAT3 (#9132), phospho-Y(705)STAT3 (#9131), phospho-S(727)STAT3 (#9134S), ERK1/2 (#9102), phosphor-T(202), Y(204)ERK1/2 (#4377), STAT1 (#9172), phospho-Y(701)STAT1 (#9171), phospho-NF-κB p65 (Ser536) (93H1) rabbit mAb (#3033) (all from Cell Signaling Technology Inc, Danvers, MA), GAPDH (#9485), NF-kB p65 (#2106) and IL-19 (#198925) (both from Abcam, Cambridge, UK) and IL-33 (ALX-804-840) (Axxora, San Diego, CA). Membranes were incubated with peroxide-conjugated secondary antibody (polyclonal swine anti-rabbit or mouse, HRP conjugated; Dako, Glostrup, Denmark) and visualized by enhanced chemiluminescence using an Amersham ECL western blotting system (GE Healthcare, Buckinghamshire, UK). For analysis bands were quantified using the Quantity One software system (Bio-Rad, Hercules, CA) and phosphorylated proteins expressed as a proportion of total protein determined from duplicate membranes.

### Arginase activity

Arginase activity was determined using the arginase assay kit according to the manufacturer’s instruction (Abnova, Taipei City, Taiwan).

### Statistical Analysis

All data were expressed as mean ± SEM and statistical analysis was performed using one-way analysis of variance (ANOVA) and the appropriate parametric or nonparametric statistical test using SigmaStat version 3.5 (Jandel Scientific, San Rafeal, CA). P values ≤ 0.05 were considered statistically significant. All authors had access to the study data and had reviewed and approved the final manuscript.

## Additional Information

**How to cite this article**: Däbritz, J. *et al.* Altered gp130 signalling ameliorates experimental colitis via myeloid cell-specific STAT3 activation and myeloid-derived suppressor cells. *Sci. Rep.* **6**, 20584; doi: 10.1038/srep20584 (2016).

## Figures and Tables

**Table 1:** Primer sequences for qPCR.

Gene	Forward Primer Sequence (5′–3′)	Reverse Primer Sequence (5′–3′)
IL-1β	CAGGCAGTATCACTCATTGTGG	GTGCAGTTGTCTAATGGGAACG
IL-6	ACAAAGCCAGAGTCCTTCAGAGA	CTGTTAGGAGAGCATTGGAAATTG
IL-10	TGATGCCCCAGGCAGAGA	CACCCAGGGAATTCAAATGC
IL-11	CTGCAAGCCCGACTGGAA	AGGCCAGGCGAGACATCA
IL-17	TGGAAGAGTATGAGCGGAACCT	GGGTCGTGGTTGATGCTGTAG
IL-19	TAAGGAGAATCAGCAGCATTGC	CACCTGACATCGCTCCAGAGA
IL-27	CCAATGTTTCCCTGACTTTCCA	AAGTGTGGTAGCGAGGAAGCA
IL-33	TATGAGTCTCCCTGTCCTGCAA	CTCATGTTCACCATCACCTTCTTC
IFNγ	TGCCACGGCACAGTCATT	CCAGTTCCTCCAGATATCCAAGA
iNOS	GGCAGCCTGTGAGACCTTTG	TGAAGCGTTTCGGGATCTG
COX2	TGCCTCCCACTCCAGACTAGA	CAGCTCAGTTGAACGCCTTTT
ARG1	AAAGCTGGTCTGTGGAAAA	ACAGACCGTGGGTTCTTCAC
S100A8	GTCCTCAGTTTGTGCAGAATATAAA	GCCAGAAGCTCTGCTACTCC
S100A9	CTCTTTAGCCTTGAAGAGCAAG	TTCTTGCTCAGGGTGTCAGG
STAT3	TTGTGATGCCTCCTTGATCGT	CTGGCAAGGAGTGGGTCTCTAG
RPL32	GAGGTGCTGCTGATGTGC	GGCGTTGGGATTGGTGACT
